# Effect of Probiotics in Stress-Associated Constipation Model in Zebrafish (*Danio rerio*) Larvae

**DOI:** 10.3390/ijms25073669

**Published:** 2024-03-25

**Authors:** Ayoung Lee, Seung Young Kim, Seyoung Kang, Seong Hee Kang, Dong Woo Kim, Jung Wan Choe, Jong Jin Hyun, Sung Woo Jung, Young Kul Jung, Ja Seol Koo, Hyung Joon Yim, Suhyun Kim

**Affiliations:** 1 Division of Gastroenterology and Hepatology, Department of Internal Medicine, College of Medicine, Korea University, Ansan 15355, Republic of Korea; frisby@korea.ac.kr (A.L.); ksymd@korea.ac.kr (S.Y.K.); dumbo83@korea.ac.kr (S.H.K.); blustick@korea.ac.kr (D.W.K.); jwchoe@korea.ac.kr (J.W.C.); sean4h@korea.ac.kr (J.J.H.); sungwoojung@korea.ac.kr (S.W.J.); 2002021168@korea.ac.kr (Y.K.J.); jskoo@korea.ac.kr (J.S.K.); gudwns21@korea.ac.kr (H.J.Y.); 2 Zebrafish Translational Medical Research Center, Korea University, Ansan 15355, Republic of Korea; rkdtpdud372@hanmail.net; 3 Department of Biomedical Sciences, College of Medicine, Korea University, Seoul 04763, Republic of Korea

**Keywords:** zebrafish, light, stress, constipation, *B. longum*

## Abstract

The pathophysiology of functional bowel disorders is complex, involving disruptions in gut motility, visceral hypersensitivity, gut–brain–microbiota interactions, and psychosocial factors. Light pollution, as an environmental stressor, has been associated with disruptions in circadian rhythms and the aggravation of stress-related conditions. In this study, we investigated the effects of environmental stress, particularly continuous light exposure, on intestinal motility and inflammation using zebrafish larvae as a model system. We also evaluated the efficacy of probiotics, specifically *Bifidobacterium longum* (*B. longum*), at alleviating stress-induced constipation. Our results showed that continuous light exposure in zebrafish larvae increased the cortisol levels and reduced the intestinal motility, establishing a stress-induced-constipation model. We observed increased inflammatory markers and decreased intestinal neural activity in response to stress. Furthermore, the expressions of *aquaporins* and *vasoactive intestinal peptide*, crucial for regulating water transport and intestinal motility, were altered in the light-induced constipation model. Administration of probiotics, specifically *B. longum*, ameliorated the stress-induced constipation by reducing the cortisol levels, modulating the intestinal inflammation, and restoring the intestinal motility and neural activity. These findings highlight the potential of probiotics to modulate the gut–brain axis and alleviate stress-induced constipation. Therefore, this study provides a valuable understanding of the complex interplay among environmental stressors, gut function, and potential therapeutic strategies.

## 1. Introduction

Functional bowel disorders, such as irritable bowel syndrome (IBS) and functional constipation, are highly prevalent conditions that increase healthcare costs and reduce the quality of life. The pathophysiology of functional bowel disorders is complex and multifactorial, involving alterations in gut motility, visceral hypersensitivity, gut–brain interactions, and psychosocial factors [1]. Trauma history or stressful events are important risk factors associated with functional bowel disorders [2]. Moreover, the concept of the microbiota–gut–brain axis has emerged in recent years, highlighting the gut microbiota’s crucial role in gut–brain communication [3]. However, many aspects of the underlying mechanisms of functional bowel disorders remain unclear, leading to a lack of definitive treatment options. Modulating the gut–brain axis, such as with probiotics, is being investigated as an attractive target for developing novel treatments [4].

Light pollution, which is a significant environmental issue, is characterized by excessive and obtrusive artificial light. Excessive exposure to light during the night is known to disrupt circadian rhythms, leading to sleep disorders, stress, and increased anxiety [5,6]. Stress can affect the digestive function, potentially leading to problems such as diarrhea and constipation [7]. In addition, irregular sleep patterns are associated with various gastrointestinal issues, including constipation.

The zebrafish presents an appealing animal model for studying the mechanisms of functional intestinal disorders, particularly the gut–brain axis, owing to its small size, high fecundity, fully annotated genome, and utility in investigating gut physiology, stress responses, and the neurologic system [8]. Typically, bowel movements are measured indirectly using the fecal pellet number or weight change, or they are analyzed via radiologic- or nuclear-imaging tests to monitor the intestinal motility over time in humans and experimental animals [9]. Other direct methods for assessing the intestinal motility include monitoring changes in the myoelectrical activity or intraluminal pressure using a transducer or catheter [10]. However, it is difficult to observe the intestinal motility in real time in vivo, and direct measurement methods generally require invasive procedures. Zebrafish (*Danio rerio*) larvae offer a promising solution as effective models for studying intestinal physiology, including gastrointestinal motility, due to their transparency and similarities in cellular structure and function to the mammalian gastrointestinal tract, despite their simple structural organization. [11]. In addition, zebrafish are also widely used to study various neurological diseases because of their resemblance to mammalians and the presence of similar neurotransmitters [12]. The zebrafish hypothalamus–pituitary–inter-renal (HPI) axis parallels the human hypothalamus–pituitary–adrenal (HPA) axis, and cortisol serves as the central stress hormone in both systems. This evolutionary link establishes the zebrafish as a valid model for cortisol-mediated stress research [13,14]. Furthermore, its gut motility is controlled primarily by the enteric nervous system, as in other vertebrates, making it a useful model for studying the gut–brain axis.

Therefore, we aimed to evaluate changes in the intestinal motility after environmental stress in zebrafish larvae, and to investigate the efficacy and related mechanism of probiotics in a stress-associated-constipation model.

## 2. Results

### 2.1. Changes in Intestinal Motility Caused by Constant-Light-Exposure Stress

**Figure 1:**
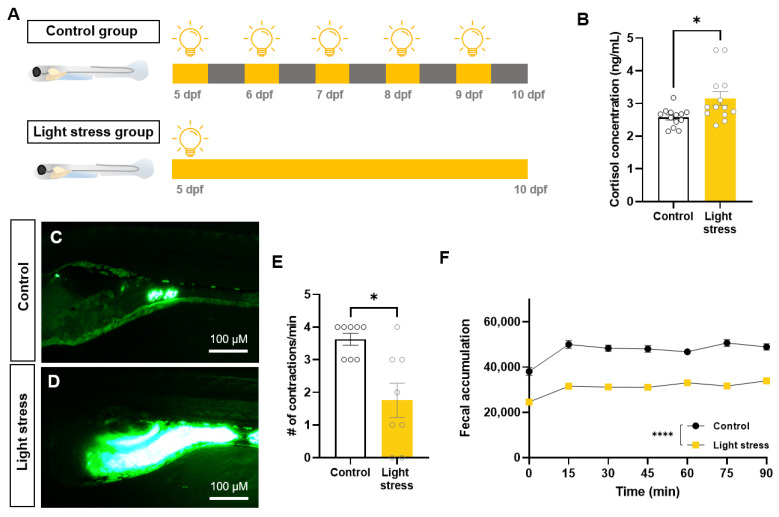
Constant light exposure causes stress and constipation in zebrafish larvae. (**A**) Schematic illustration showing experimental design. (**B**) Comparison of cortisol concentration between the control and light-stress groups (*n* = 13). (**C**,**D**) Lateral images of the fluorescent tracer in the intestinal tracts of zebrafish larvae. Scale bar: 100 μM. (**E**) Comparison of the number (#) of intestinal contractions in zebrafish larvae (*n* = 8). (**F**) Fluorescence spectrophotometer of fecal accumulation over time (*n* = 3). Each replicate had 45 zebrafish embryos. * *p* < 0.05; **** *p* < 0.0001.

To investigate the effects of environmental stress on the gastrointestinal function, we established a zebrafish model of persistent light exposure (Figure 1A). An increase in cortisol was observed in zebrafish exposed to persistent light (2.57 ± 0.28 ng/mL in control group, 3.15 ± 0.75 ng/mL in light-stress group, *p* < 0.05) (Figure 1B). The observation of larval intestines using fluorescent tracers revealed the notable presence of residual fluorescence in the light-stress group, indicating a substantial amount of tracer (Figure 1C,D). Additionally, a significant reduction in intestinal contractions was observed (3.63 ± 0.52 in control group, 1.75 ± 1.49 in light-stress group, *p* < 0.05) (Figure 1E). When the two groups were compared using a high-throughput method, fecal accumulation consistently increased in the light-stress group (Figure 1F). These results confirm that light stress induces constipation in zebrafish larvae.

### 2.2. Effect of Probiotics on Zebrafish Constipation Model Using Light Exposure

**Figure 2:**
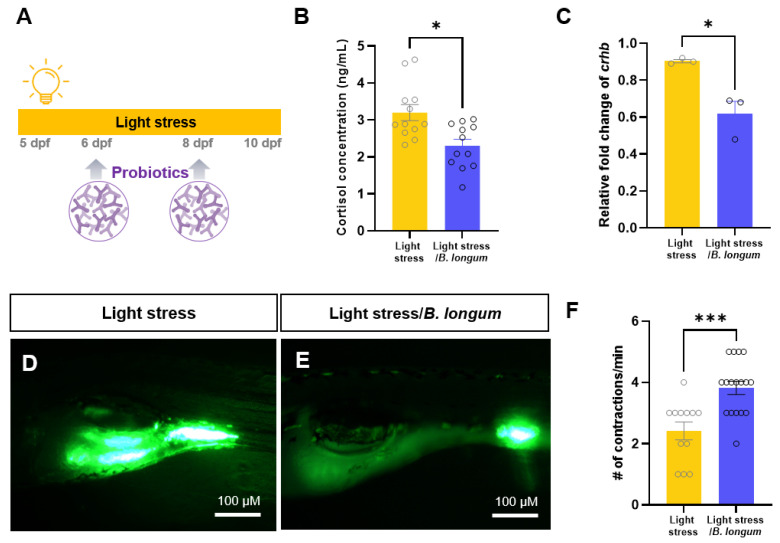
Probiotic intakes reduced stress response and constipation in zebrafish larvae. (**A**) Schematic illustration showing experimental design. Larvae were fed *Bifidobacterium longum* subsp. *longum* probiotic-based food on 6 and 8 dpf during constant light exposure. (**B**) Comparison of cortisol concentration between light-stress group and probiotic-feeding light-stress group (*n* = 12). (**C**) Relative fold change of *crhb* mRNA expression (*n* = 3). Each replicate had 20 zebrafish embryos. (**D**–**F**) Lateral images of the fluorescent tracer in the intestinal tracts of zebrafish larvae. Scale bar: 100 μM. (**F**) Comparison of the number (#) of intestinal contractions in zebrafish larvae. *n* = 12 for light-stress group, *n* = 17 for light-stress/*B. longum* group. * *p* < 0.05; *** *p* < 0.001.

To assess the effects of probiotics on a zebrafish constipation model, we initially administered two probiotics, *B. longum* and *Lacticaseibacillus rhamnosus* (*L. rhamnosus*), to a loperamide-induced-constipation model. Although the intestinal contractions were significantly reduced in the loperamide-induced-constipation model, the administration of two probiotics restored the intestinal motility to a level similar to that of the controls [15] (Supplementary Figure S1). We investigated whether probiotic intake affected stress-induced constipation. In the stress-induced-constipation model, we conducted experiments by feeding probiotics to *B. longum* every other day (Figure 2A). In non-stressed fish, there were no differences in the cortisol levels or intestinal motilities between groups with and without *B. longum* administration (Supplementary Figure S2). However, a significant decrease in the cortisol levels was noted in the stress model following probiotic administration (3.20 ± 0.75 ng/mL in light-stress group, 2.30 ± 0.61 ng/mL in light-stress/*B. longum* group, *p* < 0.05) (Figure 2B). Furthermore, probiotics significantly reduced the levels of corticotropin-releasing hormone b (*crhb*), an analog of the mammalian *CRH* gene (*p* < 0.05) (Figure 2C). Moreover, we observed an increase in intestinal contraction, resulting in the accelerated movement of the fluorescent feed (2.41 ± 0.94 in light-stress group, 3.82 ± 0.89 in light-stress/*B. longum* group, *p* < 0.001) (Figure 2D,F). These results confirmed that *B. longum* probiotics improved the stress response and increased the intestinal motility in larvae exposed to constant light.

### 2.3. Probiotic Effect on Intestinal Inflammation in Stress-Induced Constipation Model

**Figure 3:**
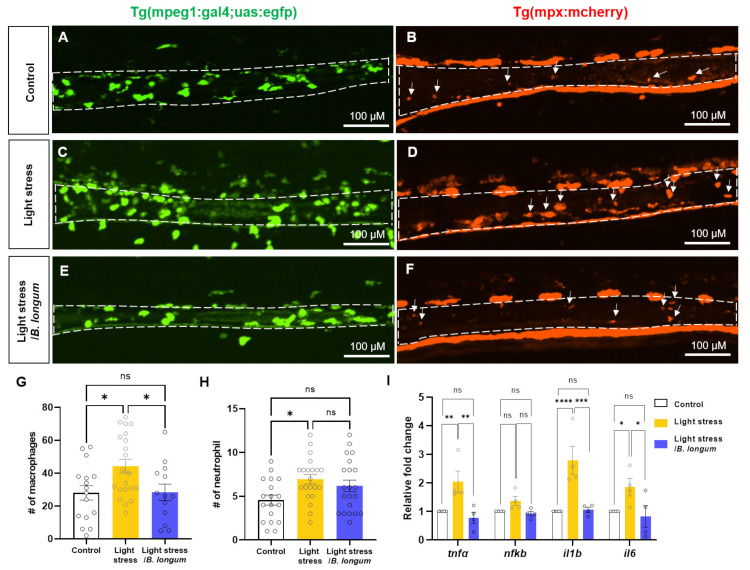
Probiotics reduced inflammatory cell recruitment by constant light exposure. (**A**,**C**,**E**) In vivo intestinal images of 10 dpf Tg (*mpeg1:gal4vp16;uas:egfp*) show macrophage infiltration. Scale bar: 100 μM. (**B**,**D**,**F**) In vivo intestinal images of 10 dpf Tg (*mpx:mcherry*) show neutrophil infiltration. Arrows indicate neutrophils in the intestine. Scale bar: 100 μM. (**A**,**B**) Control group. (**C**,**D**) Stress-induced group by constant light exposure. (**E**,**F**) *B. longum*-feeding stress-induced group. (**G**) Comparison of the number (#) of intestine-infiltrated macrophages among all groups: *n* = 16 for control group; *n* = 21 for light-stress group; *n* = 13 for light-stress/*B. longum* group. (**H**) Comparison of the number (#) of intestine-infiltrated neutrophils among all groups: *n* = 18 for control group; *n* = 21 for light-stress group; *n* = 21 for light-stress/*B. longum* group. (**I**) Relative fold changes of inflammatory genes, including *tnfα*, *nfkb*, *il1b*, and *il6* (*n* = 4). Each replicate had 20 zebrafish embryos. ns, not significant; * *p* < 0.05; ** *p* < 0.01; *** *p* < 0.001; **** *p* < 0.0001.

Next, we observed the inflammatory cells in the intestine to verify whether changes in intestinal motility are associated with intestinal inflammation and immune activation [16]. We found an increase in the intestinal macrophage infiltration in the stress-induced-constipation model using the Tg (mpeg1: gal4; uas: egfp) transgenic line. This increase was restored by probiotic administration (28.06 ± 17.5 in control group; 44.33 ± 19.1 in light-stress group; 28.38 ± 17.9 in light-stress/*B. longum* group, *p* < 0.05) (Figure 3A,C,E,G). Neutrophil infiltration, visualized using the Tg (mpx: mcherry) transgenic line, was also increased in the stress model, followed by a decreasing trend upon probiotic administration (4.56 ± 2.43 in control group; 6.95 ± 2.48 in light-stress group; 6.19 ± 3.01 in light-stress/*B. longum* group, *p* < 0.05) (Figure 3B,D,F,H). Additionally, inflammatory cytokines, such as *tnfα*, *il1b*, and *il6*, were statistically significantly elevated in the stress model and showed a subsequent recovery to lower levels similar to those in the control group after probiotic administration (Figure 3I). A similar change was observed in the *nfkb* expression levels, although the difference was not statistically significant. These results indicate that light stress triggers intestinal inflammation, which is associated with constipation.

### 2.4. Probiotic Effect on Intestinal Folds in Stress-Induced Constipation Model

**Figure 4:**
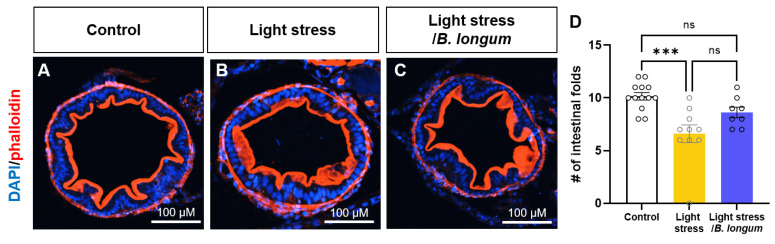
Inflammation induced by stress affects intestinal folds. (**A**–**C**) Cross-sectional images of 10 dpf larvae labeled with phalloidin (red) and DAPI (blue). Scale bar: 100 μM. (**D**) Comparison of the number (#) of intestinal folds of mid-intestines among all groups: *n* = 13 for control group; *n* = 10 for light-stress group; *n* = 8 for light-stress/*B. longum* group. ns, not significant; *** *p* < 0.001.

Because inflammation is known to cause a loss of intestinal folds, we observed reduced intestinal folds in the constant-light-exposure-stress-induced group. Using phalloidin staining, an F-actin marker, we observed a decrease in the number of fold changes in the stress group (10.15 ± 1.28 in control group, 6.6 ± 2.67 in light-stress group, *p* < 0.05) (Figure 4A,B,D). There was a slight recovery of the observed alterations after probiotic administration; however, this increase was not statistically significant (8.625 ± 1.41 in light-stress/*B. longum* group, *p* = 0.31) (Figure 4C,D).

### 2.5. Probiotic Effect on Enteric Neural Activity in Stress-Induced Constipation Model

**Figure 5:**
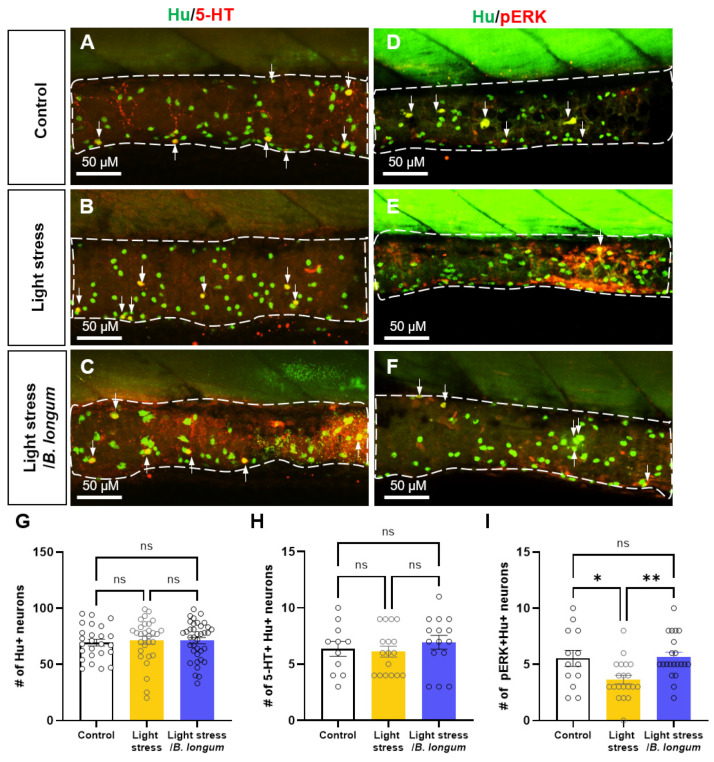
Inflammation-induced activity of enteric neurons. (**A**–**C**) Intestinal images of 10 dpf larvae labeled with anti-Hu (green) and anti-5-HT (red) antibodies. Arrows indicate 5-HT+/Hu+ neurons in the intestine. Scale bar: 50 μM. (**D**–**F**) Intestinal images of 10 dpf larvae labeled with anti-Hu (green) and anti-pERK (red) antibodies. Arrows indicate pERK+/Hu+ neurons in the intestine. Scale bar: 50 μM. (**G**) Comparison of the number (#) of Hu+ enteric neurons among all groups: *n* = 25 for control group; *n* = 30 for light-stress group; *n* = 38 for light-stress/*B. longum* group. (**H**) Comparison of the number (#) of 5-HT+/Hu+ serotonin enteric neurons among all groups: *n* = 12 for control group; *n* = 17 for light-stress group; *n* = 15 for light-stress/*B. longum* group. (**I**) Comparison of the number (#) of pERK+/Hu+ enteric neurons among all groups: *n* = 13 for control group; *n* = 20 for light-stress group; *n* = 21 for light-stress/*B. longum* group. ns, not significant; * *p* < 0.05; ** *p* < 0.01.

To determine whether changes in the intestinal motility were influenced by intestinal neurons, we examined the expressions of the serotonergic neurotransmitter 5-HT and the neuronal activity marker pERK using the pan-neuronal marker Hu. The number of Hu+ enteric neurons did not change after stress induction or probiotic administration (70.6 ± 18.31 in control group; 81.5 ± 8.89 in light-stress group; 71.35 ± 16.9 in light-stress/*B. longum* group, *ns*) (Figure 5A–G). In addition, no significant difference was observed in the serotonergic enteric neurons (5-HT+ Hu+) between the stress-induced-constipation model and the probiotic-fed constipation group (6.36 ± 2.20 in control group; 6.12 ± 1.99 in light-stress group; 6.93 ± 2.40 in light-stress/*B. longum* group, *ns*) (Figure 5A–C,H). However, the activity of the enteric neurons (pERK+ Hu+) was significantly decreased in the light-stress-induced-constipation group (Figure 5D,E,I). The decreased neural activity was restored upon probiotic administration (5.17 ± 2.33 in control group; 3.63 ± 1.77 in light-stress group; 5.55 ± 1.88 in light-stress/*B. longum* group, *p* < 0.05 and *p* < 0.01 each, respectively) (Figure 5F,I). These results suggest that probiotics regulate intestinal motility by influencing the enteric neuronal activity.

### 2.6. Stress-Induced-Constipation and Probiotic-Administration-Induced Changes in the Expressions of Aquaporins and Vasoactive Intestinal Peptide (vip)

**Figure 6:**
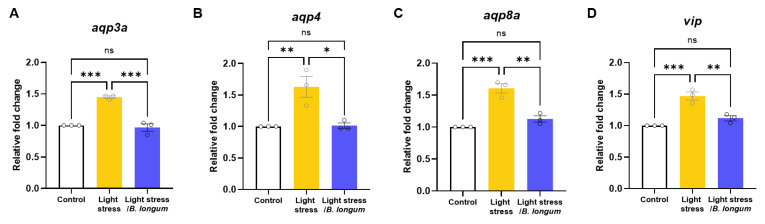
Probiotics relieve stress-induced constipation by affecting intestinal water metabolism. Relative fold changes in *aqp3a* (**A**), *aqp4* (**B**), *aqp8a* (**C**), and *vip* (**D**) mRNA expressions (*n* = 3). Each replicate had 20 zebrafish embryos. ns, not significant; * *p* < 0.05; ** *p* < 0.01; *** *p* < 0.001.

Because intestinal dehydration is a common cause of constipation, we examined the changes in the expressions of genes involved in water transport. We observed an increase in *aquaporin-3*, -*4*, and -*8* mRNA in the stress-induced-constipation model, which was significantly decreased after probiotic administration (Figure 6A–C). Additionally, the level of *vip*, a regulator of ion and water transport, as well as intestinal motility, increased in the stress-induced constipation model and decreased after probiotic administration (Figure 6D). These data indicate that stress can cause constipation by affecting the expressions of genes that regulate water transport in the intestine. 

## 3. Discussion

The activation of neurophysiological pathways that worsen constipation symptoms can be influenced by adverse and stressful life experiences [17]. To study constipation by stress, we established a stress-induced-constipation model in zebrafish by employing environmental stress through light/dark-cycle changes without a dark cycle. Maintaining the light/dark cycle to uphold a normal circadian clock is important for the growth and development of zebrafish larvae [18]. We showed that continuous light exposure elevated the cortisol levels and reduced the intestinal motility in the zebrafish larvae. Our findings were supported by previous studies in which stress altered the gastrointestinal (GI) motility and induced changes in the secretions, intestinal barrier permeability, and gut microbiota [7]. A study on mice subjected to water-immersion stress reported a reduction in longitudinal contractions of both the proximal and distal colon [19]. Restrained C57BL/6 mice exhibited a diarrhea-like phenotype by increased motility [20]. Further research is needed, as the effects on the gut motility may vary depending on the type and duration of the stress and differences in the species tested. 

Our investigation revealed an increase in the number of inflammatory cells and expressions of cytokine genes following stress. IBS, a well-known GI disorder associated with stress, is characterized by low-grade inflammation, with increased numbers of mast cells and inflammatory cytokines observed in patients compared with healthy individuals [21]. Recent studies have shown a growing interest in muscular macrophages among the intestinal macrophage subtypes. Although conventional macrophages trigger an inflammatory response to bacterial stimuli, muscular macrophages exhibit unique phenotypic profiles and cytokine production that restrain responses to bacterial infections, thereby protecting the gut from excessive inflammation [22,23]. In addition, muscular macrophages collaborate with the enteric nervous system to regulate gut secretion and motility. Alterations in the intestinal macrophage function and abundance may contribute to the development of various gastrointestinal diseases [22]. Although our study did not distinguish among the macrophage subtypes, it is plausible that intestinal macrophages may have contributed to the regulation of the intestinal motility.

Current guidelines for chronic constipation recommend dietary interventions, such as modifying fiber intake as the initial treatment, followed by laxatives [23,24]. However, dissatisfaction with the traditional treatment methods is reported by half of the individuals with chronic constipation, mainly owing to a perceived lack of efficacy [25]. A meta-analysis of 30 randomized controlled trials revealed that probiotics improved the stool frequency in patients with constipation [26]. Another meta-analysis focusing on probiotic effects in patients with constipation-predominant IBS also demonstrated an enhancement in the stool consistency scores [27]. We demonstrated that the administration of *B. longum* improved the intestinal motility and reduced the inflammatory responses in a stress-induced-constipation zebrafish model. Consistent with our findings, *B. longum* administration restored the increased inflammatory response and decreased the intestinal motility in the aluminum sulfate- and TNBS-treated zebrafish model [28,29]. Therefore, this study supports the therapeutic effect of probiotics in constipation.

The gut–brain axis is a bidirectional interaction channel between the gastrointestinal tract and central nervous system. Probiotics may influence these interactions by sending signals to the brain that modulate stress and emotional responses [30]. The relationship between probiotics and stress response reduction is an area of ongoing research, but the exact mechanisms are not yet fully understood. However, some studies have suggested that probiotics, beneficial bacteria that promote a healthy gut microbiota, may have a positive impact on stress and anxiety [31]. In the stress group, the administration of *B. longum* resulted in improved bowel movements and decreased cortisol levels. These findings demonstrate the potential of the gut–brain axis in modulating the stress response by affecting the gut microbiome with probiotics.

In this stress-induced-constipation model, we observed a decrease in neural activity, as indicated by the pERK expression, which was recovered after probiotic administration, suggesting a role for enteric neurons in enhancing the intestinal motility via probiotics. The decreased activity or dysfunction of the enteric neurons that control muscle contractions can slow the movement of waste through the intestines, leading to constipation [32,33]. However, other studies have shown increased pERK expression in dorsal horn neurons during stress or tissue injury, along with ERK involvement in neural and synaptic plasticity associated with pain hypersensitivity [34,35]. These findings suggest a discrepancy with our current results regarding the intestinal stress response, indicating a need for future investigations into the neural activity in neurons of the central nervous system.

Aquaporins (AQPs) are essential proteins for maintaining fluid homeostasis and facilitating the transmembrane transport of water, glycerol, and small solutes. They regulate the intestinal water absorption and secretion, thereby ensuring a balance between the fluid movement and environmental homeostasis [36]. The abnormal expression of aquaporins in intestinal tissues, whether overexpressed or underexpressed, can disrupt the intestinal balance, leading to conditions such as diarrhea or constipation [37]. The high expression of AQPs can lead to excessive water transport from the lumen to the blood vessels, resulting in hardened feces and constipation. In our stress-induced-constipation model, we confirmed an increase in the aquaporin mRNA expression, which was restored by probiotic administration. These results support previous studies showing that probiotics improved the intestinal barrier function and regulated the intestinal fluid balance [38]. In a rat model of loperamide-induced constipation, probiotic administration increased the fecal water content and intestinal motility by modulating gastrointestinal peptides such as motilin, vasoactive intestinal peptide (VIP), and AQP3 [39].

## 4. Materials and Methods

### 4.1. Reagents

FluoSpheres™ Carboxylate-Modified Microspheres were purchased from Thermo Fisher Scientific (Waltham, MA, USA). Tricaine (MS-222), dimethyl sulfoxide (DMSO), and loperamide were purchased from Sigma-Aldrich (St. Louis, MO, USA). Probiotic types *Bifidobacterium longum* subsp. *Longum* (KCTC 3128) and *Lacticaseibacillus rhamnosus* (KCTC 3237) were obtained from the Korean Collection for Type Cultures (KCTC).

### 4.2. Zebrafish Maintenance

Wild-type zebrafish, Tg (mpeg1:gal4;uas:egfp) [40], and Tg (mpx:mcherry) [41] were used in this study. Adult zebrafish were housed in light- and temperature-controlled aquaculture facilities with a standard 14 h light/10 h dark photoperiod. All the experiments were approved by the Korea University Institutional Animal Care and Use Committee (No. KOREA-2023-0200).

### 4.3. Fluorescent Tracer

The fluorescent tracer was prepared by mixing 100 mg of powdered larval feed (O.range start-s, INVE aquaculture, Salt Lake City, UT, USA) with 150 µL of yellow-green 2.0 µL of FluoSpheres Carboxylate-Modified Microspheres (Thermo Fisher, F8827) and 50 µL of deionized water. The mixture was dried overnight at 25 °C in the dark and crushed into a fine powder.

### 4.4. Probiotic-Based Feeding

Feeding was performed through the administration of probiotic-based food twice a week. Based on the previous experiment [42], the formulation for the preparation of the probiotic-based food was as follows: 1.5 g skimmed milk (BD Difco, Sparks, MD, USA), 30 g larval feed, and 1 g probiotics (*Bifidobacterium longum* subsp. *Longum* and *Lacticaseibacillus rhamnosus*).

### 4.5. Stress Induction with Environmental Stimuli

The light-stress group was raised under a continuous-light photoperiod from 5 to 10 dpf. Light stress was induced by housing with a light photoperiod for 24 h without a dark photoperiod for a certain period. The control group was raised under a standard 14 h light/10 h dark photoperiod.

### 4.6. Cortisol Assay

Cortisol was extracted as follows: 25–30 frozen zebrafish per group were added to 1 mL of phosphate-buffered saline (PBS) and homogenized. The mixture was vortexed for 1 min with 5 mL of diethyl ether (Sigma-Aldrich, 296082). The mixture was centrifuged at 3000× *g* for 10 min. The ether was dried in a 45 °C water bath for more than 1 h, and then 1 mL of PBS was added. Cortisol levels were quantified using a Cortisol Parameter Assay Kit (R&D Systems, KGE008B, Minneapolis, MN, USA).

### 4.7. Quantitative Reverse Transcription Polymerase Chain Reaction (RT-qPCR)

Total RNA was extracted from the zebrafish larvae using TRizol reagent (Molecular Research Center, TR118, Cincinnati, OH, USA) according to the manufacturer’s protocol. The RNA concentration was determined using a Nanodrop spectrophotometer (Nanodrop Technologies, ND-1000, Wilmington, DE, USA). Complementary DNA (cDNA) was synthesized from 1 μg of total RNA using Enzynomics TOPscript RT DryMIX (Enzynomics, RT200, Daejeon, Republic of Korea). Relative gene expression was quantified via RT-PCR using Power SYBR Green PCR Master Mix (Thermo Fisher, Applied Biosystems, 43-676-59, Waltham, MA, USA) according to the manufacturer’s protocol. The reaction mixture (25 µL) consisted of Power SYBR Green PCR Master Mix, 10 pmol of primer pairs, and 1 μL of the cDNA as a template. Primer sequences are summarized in Table 1. The PCR reaction was performed at 95 °C for 10 min, followed by 40 cycles at 95 °C for 10 s, an annealing temperature for 30 s, and 72 °C for 30 s. After 40 cycles, a melting step was performed at 60 °C for 60 s, followed by 95 °C for 15 s. RT-PCR was performed using the QuantStudio5 (Applied Biosystems, Waltham, MA, USA). All PCRs were normalized to beta-actin expression to control for variations.

### 4.8. High-Throughput Measurement of Gut Transit Time

High-throughput measurement of the gut transit time using larval zebrafish was performed as described previously [47]. After larvae were allowed to feed on fluorescent food for 1 h, they were rinsed three times with embryo medium (EM,: 15 mM: NaCl;: 0.5 mM ; KCl; : 1 mM; CaCl_2_; : 1 mM; MgSO_4_;: 0.15 mM; KH_2_PO_4_;: 0.05 mM; NH_2_PO_4_;: 0.7 mM NaHCO_3_). The larvae were then withdrawn with 100 μL embryonic medium and distributed into wells in 96-well plates. The 96-well-plates were placed in a plate spectrophotometer. For the yellow-green label, the appropriate wavelengths of light were excited at 505 nm and detected at 515 nm. The fluorescence of the 96-well-plate was read five times from the bottom five in immediate succession without shaking the plate. The minimum value of the five readings per well and the initial fluorescence from each well were used. Fluorescence in the 96-well plates was read at 15 min intervals for 90 min.

### 4.9. Peristaltic-Movement Measure

Stress-induced 10 dpf larvae were fed normal food or probiotic-based food for 1 h and were then washed three times with embryo medium. Loperamide-induced constipation was achieved by incubating the larvae in 5 mg/mL loperamide for 2 h. They were anesthetized with 0.2% tricaine, and the number of intestinal movements per minute was measured using a fluorescence microscope.

### 4.10. Immunohistochemistry

Zebrafish larvae were fixed with 4% paraformaldehyde and washed thrice with PBT/0.5% Triton X-100. Larvae were permeabilized sequentially with acetone (7 min at −20 °C), 0.25% trypsin/PBS (HyClone, SH30042, Logan, UT, USA) (10 min at −20 °C), and 1 mg/mL collagenase (Sigma-Aldrich) (40–90 min). Then, larvae were blocked in 10% bovine serum albumin (BSA) with 6% sheep serum and subsequently treated with anti-rabbit 5-HT (Sigma Aldrich, 1:250), anti-phospho p44/42 MAP kinase (Thr202/Tyr204) (pERK) (Cell signaling, Danvers, MA, USA, 1:250), and anti-mouse Hu (Invitrogen, Waltham, MA, USA, 1:100) overnight at 4 °C. Alexa Fluor 488- and 568-conjugated secondary antibodies (1:500; Invitrogen) were used to detect the primary antibodies. For α-actin labeling, 10 μm frozen-section samples were labeled with Phalloidin-iFluor 594 (abcam, Cambridge, UK, 1:1000) and then counterstained with 40, 6-diamidino-2-phenylindole, dihydrochloride (DAPI) (D1306, Thermo Fisher Scientific).

### 4.11. Statistical Analysis

All statistical analyses were performed using GraphPad Prism version 9 (GraphPad Software Inc., La Jolla, CA, USA). For comparisons between two groups, the Student’s unpaired *t*-test and Mann–Whitney U test were performed according to normality. For comparisons among multiple groups, one- and two-way ANOVA and the Kruskal–Wallis test with post hoc analysis were performed according to normality. All data are expressed as means ± standard errors of the means (SEMs). Statistical significance was set at * *p* < 0.05, ** p < 0.01, *** p < 0.001, and **** p < 0.0001.

## 5. Conclusions

We established a stress-associated-constipation model using zebrafish, and we elucidated the effects and underlying mechanisms of probiotics in this model. Our findings highlight the role of the gut–brain axis in regulating the stress response and gastrointestinal function. Further investigation of the communication between gut microbes, enteric neurons, and the central nervous system is needed. In conclusion, this study sheds light on the complex interactions between environmental stressors, the gut function, and probiotic therapy in the context of constipation. By presenting the baseline data, our findings pave the way for the development of innovative treatments for constipation and other stress-related functional bowel disorders.

## Figures and Tables

**Table 1:** Primers used in this study.

Gene	Forward Primer (from 5′ to 3′)	Reverse Primer (from 5′ to 3′)	NCBI Accession Number	Reference
*β-actin*	ACCCAGACATCAGGGAGTG	CATCCCAGTTGGTCACAATAC	NM_131031	Yang J et al., 2021 [43]
*crhb*	TGAATGTAGAGCCATCGAGAGCAG	TGCCGAGCCGGATGAAGTAC	NM_001007379	
*il1b*	TGGACTTCGCAGCACAAAATG	GTTCACTTCACGCTCTTGGATG	NM_212844	Yang J et al., 2021 [43]
*il6*	ATCCGCTCAGAAAACAGTGCT	GTCGCCAAGGAGACTCTTTAC	NM_001261449	Yang MJ et al., 2020 [44]
*tnf-α*	ATAAGACCCAGGGCAATCAAC	CAGAGTTGTATCCACCTGTTAAATG	NM_212859	Yang J et al., 2021 [43]
*nf-kb*	GCTCATTCAGATTGCTCTACAC	CGTGTCTCCGTTCTCATCT	NM_001001840	Yang J et al., 2021 [43]
*aqp3a*	GCAGACTTTCCAAATCCGCAACAAG	GAACCACAGCCAAACATCACCAG	NM_213468	Ahi EP et al., 2022 [45]
*aqp 4*	AAGCGTAATGATCTCAAAGGTT	TGGAGAGGACGTCAGCATAG	NM_001003749	Grupp L et al., 2010 [46]
*aqp8a*	GTCAACTTGCTCCCTTCTGC	GGCTCGTGCAGGATTCATAC	NM_001004661	
*vip*	CGGGCTCTTCACAAGCGGAT	CTTCATCGGCGCCTGGTCTT	NM_001114553	

## Data Availability

Data supporting the findings of this study are available in the article and Supplementary Information files or from the corresponding authors upon reasonable request.

## Supplementary Materials

The following supporting information can be downloaded at https://www.mdpi.com/article/10.3390/ijms25073669/s1.
