# Supplementary material for: Effect of Probiotics in Stress-Associated Constipation Model in Zebrafish (Danio rerio) Larvae
Source: Int J Mol Sci. 2024 Mar 25;25(7):3669. doi: 10.3390/ijms25073669 (PMC11012156; doi:10.3390/ijms25073669)
Supplement: Supplementary file 1 [file ijms-25-03669-s001.zip › ijms-2904856-supplementary.pdf]

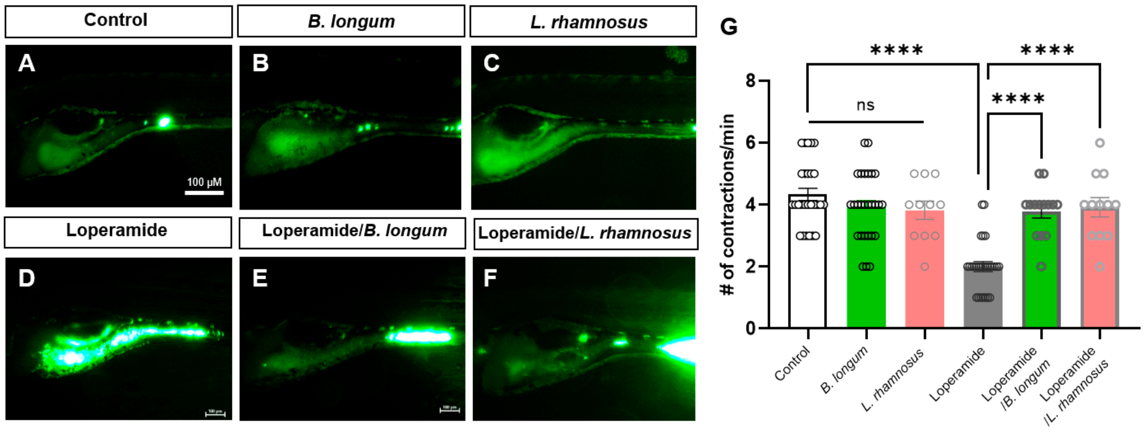

Figure S1. Probiotics improves intestinal motility in loperamide-induced constipation.

(A-F) Lateral images of the fluorescent tracer in the in the intestinal tract of zebrafish larvae. Scale bar: 100 µm. (G) Comparison of the number of intestinal contraction in zebrafish larvae. n=27 for control, *B.longum*, loperamide group. n=11 for *L. rhamnosus* group. n=14 for loperamide/ *B.longum*, n=12 for loperamide/ *L. rhamnosus* group. One-way ANOVA with Tukey's multiple comparison test. ns, not significant; ****, $p < 0.0001$.

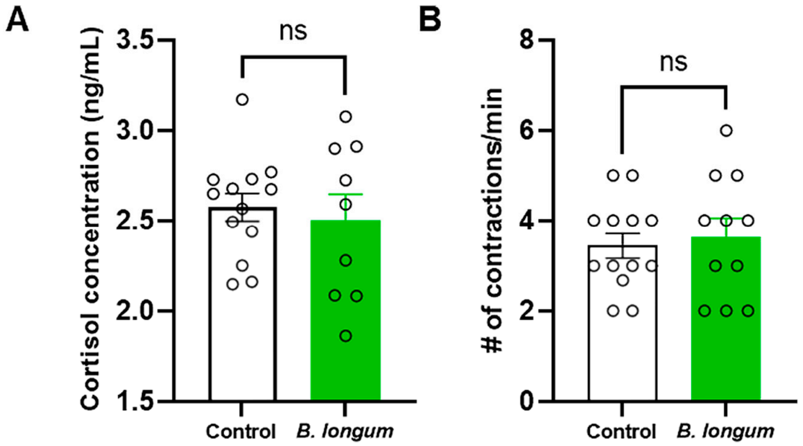

Figure S2. The effect of *B.longum* on non-stressed zebrafish larvae (A) Comparison of cortisol concentration between the non-stressed fish fed with B. *longum* and those fed without *B. longum.* n=13 for Control, n=9 for *B.longum* (B) Comparison of the number of intestinal contractions in zebrafish larvae. n=13 for Control, n=11 for *B.longum*. ns, not significant.
